# How to Structure a Successful Organ Donation and Transplantation System in Eight (Not So Easy) Steps: An Italian Case Study

**DOI:** 10.3389/ti.2023.11010

**Published:** 2023-05-25

**Authors:** Jasmine Mah, Charlotte Johnston-Webber, Apostolos Prionas, Jacopo Romagnoli, Simon Streit, George Wharton, Elias Mossialos, Vassilios Papalois

**Affiliations:** ^1^ Department of Medicine, Dalhousie University, Halifax, NS, Canada; ^2^ Department of Health Policy, London School of Economics and Political Science, London, United Kingdom; ^3^ Department of Surgery, Imperial College, London, United Kingdom; ^4^ Department of General Surgery, Whipps Cross Hospital, Barts Health NHS Trust, London, United Kingdom; ^5^ Dipartimento di Scienze Mediche e Chirurgiche, Unita’ Operativa Complessa Trapianti di Rene, Fondazione Policlinico Universitario A. Gemelli Istituto di Ricovero e Cura a Carattere Scientifico (IRCCS), Rome, Italy; ^6^ Dipartimento di Medicina e Chirurgia Traslazionale, Università Cattolica del Sacro Cuore, Rome, Italy; ^7^ Institute of Global Health Innovation, Imperial College, London, United Kingdom; ^8^ Renal and Transplant Unit, Hammersmith Hospital, Imperial College Healthcare NHS Trust, London, United Kingdom

**Keywords:** organ transplantation, Italy, the Italian transplant network, Centro Nazionale Trapianti, organ donation, transplantation policy, transplant system

## Abstract

Valuable information can be obtained from a systematic evaluation of a successful national transplant program. This paper provides an overview of Italy’s solid organ transplantation program which is coordinated by the National Transplant Network (Rete Nazionale Trapianti) and The National Transplant Center (Centro Nazionale Trapianti). The analysis is based on a system-level conceptual framework and identifies components of the Italian system that have contributed to improving rates of organ donation and transplantation. A narrative literature review was conducted and the findings were validated iteratively with input from subject matter experts. The results were organized into eight critical steps, including 1) generating legal definitions of living and deceased donation, 2) taking steps to ensure that altruistic donation and transplantation become part of the national culture and a point of pride, 3) seeking out existing examples of successful programs, 4) creating a situation in which it is easy to become a donor, 5) learning from mistakes, 6) working to diminish risk factors that lead to the need for organ donation, 7) increasing the rate of donations and transplantations *via* innovative strategies and policies, and 8) planning for a system that supports growth.

## Graphical Abstract

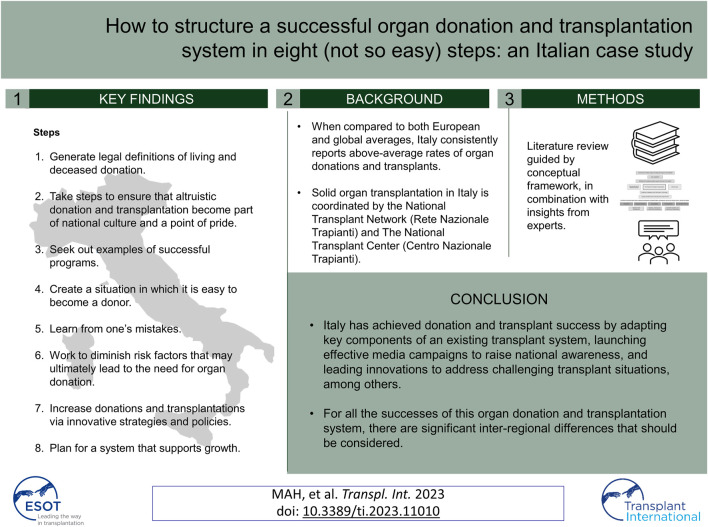

## Introduction

The worldwide increase in the average age of the population suggests that there will be a parallel increase in the number of adults living with chronic medical conditions and that the need for solid organ transplantation will remain high. Thus, there is currently significant interest focused on ways to improve the efficiency and equity of the existing national transplantation systems. Valuable lessons can be learned from countries with successful transplant programs because many of the factors that contribute to their success might be adopted by other jurisdictions and altered to suit their specific contexts and cultures.

Italy has developed one of the most successful organ transplant programs in Europe (1). In 2019, Italy was among the top ten countries in terms of the number of deceased donation per million population (1). When compared directly to both European and global averages, Italy consistently reports above-average rates of organ donations and transplants, at 21.54 and 57.9 per million population (pmp), respectively (2). Solid organ transplantation in Italy is coordinated by the National Transplant Network (Rete Nazionale Trapianti) and The National Transplant Center (Centro Nazionale Trapianti [CNT]), which is a technical and scientific organization within the Ministry of Health (Istituto Superiore di Sanita [ISS]) that operates within the Italian National Health Service (Servizio Sanitario Nazionale [SSN]) (3, 4).

Italy has a population of 59.6 million (5) and healthcare is provided by the Italian National Health Service (Servizio Sanitario Nazionale—SSN), which established a tax -based universal healthcare system in 1978 (6). In 2021, Italy spent EUR 2525 *per capita* on healthcare. This represented 8.7% of gross domestic product (GDP). In the same year the European Union (EU) average was 9.9% of GDP, or EUR 3523 *per capita* (5). Italy has one of the highest life expectancies in Europe, at 82.4 years (5) Similar to many other high-income countries, the Italian population is ageing and one of the main drivers of morbidity and mortality in the Italian population is the high burden of cardiovascular diseases (CVD). The prevalence rate of cardiovascular disease was 5,099 per 100,000 persons for men and 3,975 per 100,000 persons for women in 2015 (7). There is also high prevalence of hypertension (52% for men and 38% for women); dyslipidaemia (35% for men and 37% for women); and diabetes (12% in men and 8% in women) (8). Smoking, dietary behaviours, alcohol consumption and low physical activity are key health risk factors in Italy. In particular, smoking in Italy is above the EU average for adults and for adolescents (5). Key health system and health status information is summarized in Table 1.

**TABLE 1:** Health system financing and population health in Italy: key statistics.

Health system	References
• Tax-based universal healthcare system provided by the Italian National Health Service [Servizio Sanitario Nazionale (SSN)]	
• Health spending *per capita*, EUR 2525; EU average, EUR 3523	(5)
• Health spending as a percentage of the gross domestic product, 8.7%; EU average, 9.9%	(5)
• Public spending as a percentage of total healthcare expenditure, 74%; EU average, 79.7%	(5)
• Out-of-pocket payments as a percentage of total healthcare expenditure, 23%; EU average, 15.4%	(5)
• Percentage of the population reporting an unmet need for medical care, 1.8%; EU average, 1.7%	(5)
Health status	
• Percentage of the population over 65 years of age, 23.2%; EU average, 20.6%	(5)
• Life expectancy, 82.4 years; EU average, 80.6 years	(5)
• Percentage of adults that smoke daily, 19%; OECD average, 16.5%	(5)
• Liters of alcohol consumed *per capita*, 8L; OECD average, 8.7L	(5)
• Percentage of adults that are overweight or obese (BMI >25), 46%; OECD average, 56.4%	(5)
• Individuals maintained on renal replacement therapy incidence, 165 pmp; prevalence, 1,276 pmp	(5)

EUR, euro; EU, European Union; OECD, Organisation for Economic Co-operation and Development; BMI, body mass index.

The organ donation and transplantation program in Italy was modelled on the highly successful program developed in Spain. The Italian organ donation and transplantation program consistently achieves above-average rates of both organ donation and transplantation compared to EU member states as well as globally (Table 2). This paper will examine the main features of the Italian program with a focus on the factors that have enabled it to become one of the best organ donation and transplantation programs in the EU.

**TABLE 2:** Numbers and rates of organ donation and transplantation in Italy in 2019 compared to European Union (EU) and global averages from the Global Observatory on Donation and Transplantation (2).

	Italy	Europe	Global
Actual Deceased Donation (DD)	1,495 (25.25)	13,397 (16.47)	41,695 (6.97)
Actual DD After Brain Death (DBD)	1,415 (23.9)	11,242 (13.82)	32,453 (5.43)
Actual DD After Circulatory Death (DCD)	80 (1.35)	2,155 (2.65)	9,242 (1.55)
Total Kidney Transplants	2,139 (36.13)	28,189 (34.66)	102,539 (17.15)
Deceased Kidney Transplants	1,799 (30.39)	20,300 (24.96)	64,104 (10.72)
Living Kidney Transplants	340 (5.74)	7,889 (9.7)	38,435 (6.43)
Total Liver Transplants	1,301 (21.98)	10,794 (13.27)	36,785 (6.15)
Deceased Liver Transplants	1,277 (21.57)	8,969 (11.03)	28,137 (4.71)
Living Liver Transplants	24 (0.41)	1,808 (2.22)	7,644 (1.28)
Heart Transplants	245 (4.14)	2,862 (3.52)	8,857 (1.48)
Lung Transplants	153 (2.58)	2,331 (2.87)	6,811 (1.14)
Pancreas Transplants	42 (0.71)	763 (0.94)	2,352 (0.39)
Small Bowel Transplants	1 (0.02)	39 (0.05)	146 (0.02)
Total Organ Transplants	3,881 (65.56)	44,978 (55.3)	157,490 (26.34)

Data shown are absolute numbers followed by number per million population (pmp) in parentheses; (−) data not available or not applicable.

## Materials and Methods

Using a conceptual systems framework that addresses the essential elements of a successful system (9), our goal was to determine which components have contributed directly to the increasing rates of organ donation and transplantation reported in Italy. Our analysis will also highlight system innovations that may be useful for countries intending to establish their own transplant systems.

An assessment of the current state of Italy’s organ donation and transplantation program was performed based on the aforementioned framework criteria *via* a narrative review of the literature, complemented by interviews with a panel of international experts in transplantation and health systems. This group included one expert specifically from the Italian program. The literature review was conducted *via* a search of the Medline and Web of Science databases. We identified peer-reviewed publications using the keywords “organ donation and transplantation” and “Italy.” The search excluded publications that were written in languages other than English or Italian. We also conducted hand searches of the references listed in the original set of studies that were retrieved. One researcher (JM) screened the titles and abstracts and made selections based on their relevance to the study objectives. Grey literature was an important source of information on this topic. These sources included Google Scholar, government websites (Italian National Institute of Health [ISS]), and websites maintained by key international organ donation and transplantation organizations (e.g., Eurotransplant and the European Directorate for the Quality of Medicines).

**FIGURE 1:**
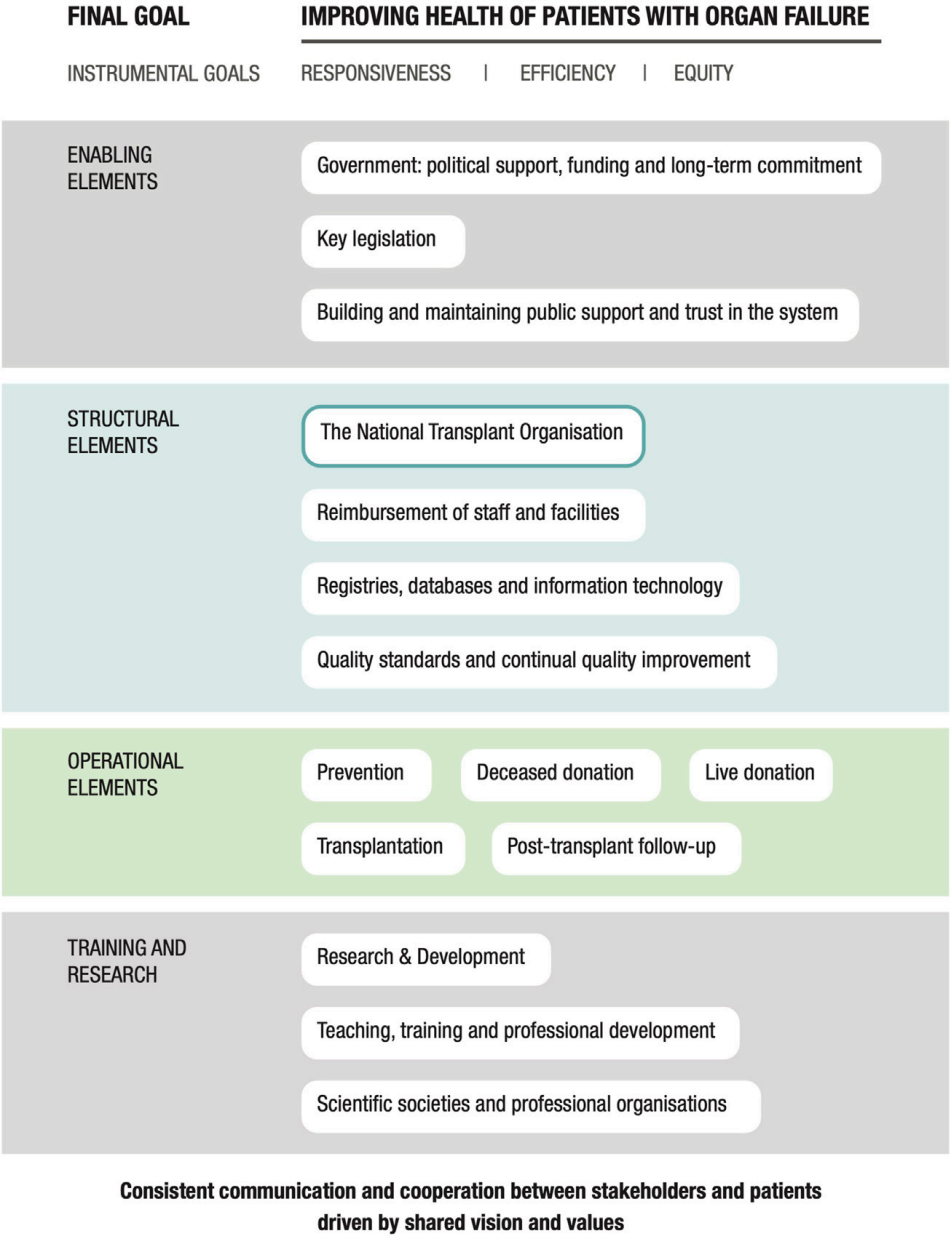
A conceptual framework for evaluating national solid organ donation and transplantation programs: the essential building blocks.

The findings were organized and coded based on a conceptual systems framework as shown in Figure 1 (9). Similar to the data collection, this information was generated in multiple steps following an iterative process in which the essential building blocks of this conceptual framework were used to guide the organization of findings that were pertinent to the Italian system. All information was validated by the expert panel and checked for any inconsistencies or misrepresentations. The results are categorized according to the domains of the conceptual framework and in terms of the most important insights derived from the analysis of the Italian program. The analysis focused on structures, processes and distinctive features of the system corresponding to domains of the framework, rather than performance in relation to health outcomes or health system goals.

## Results

The following sections present the main findings of the study and highlight the key components of the Italian organ donation and transplantation program that have contributed to its success. The findings are organized according to the domains of the conceptual framework and presented as eight steps that might be undertaken to achieve a similarly successful program (Table 3).

**TABLE 3:** Essential components of the Italian organ donation and transplantation program.

Framework Domain	Critical Steps	Details
Enabling Elements: Government: Key Legislation	Step 1: Generate legal definitions of living and deceased donation	Clear legislation focused on issues including controlled and uncontrolled donation after circulatory death, LD, brain death, and the structure of the National Transplant Organization (NTO) served to legitimize the program
Enabling Elements: Building and Maintaining Public Support and Trust in the System	Step 2: Take steps to ensure that altruistic donation and transplantation become part of the national culture and a point of pride	Affecting personal stories featured in sustained media and educational campaigns helped to change attitudes and raise the profile of the organ donation and transplantation program
Structural Elements: The National Transplant Organisation; Infrastructure; Reimbursement of Staff and Facilities	Step 3: Seek out examples of successful programs	Italy has modeled critical components of its system on the program that existed in Spain and adapted them successfully to its own national setting
Structural Elements: Registries, Databases and Information Technology	Step 4: Create a situation in which it is easy to become a donor	A “soft opt-out” policy together with multiple opportunities to register as an organ donor have helped to boost donation rates
Structural Elements: Quality Standards and Continual Quality Improvement	Step 5: Learn from one’s mistakes	Mistakes may be perceived as opportunities for learning and can be used to optimize quality and safety
Operational Elements: Prevention	Step 6: Work to diminish risk factors that lead to the need for organ donation	Prevention of organ failure is a national priority. Italy has successfully established several public health and screening programs that address this issue in the primary care setting
Operational Elements: Donation; Transplantation	Step 7: Increase donations and transplantations via innovative strategies and policies	Italy has innovative split liver programs and is a leader in developing novel perfusion techniques. The first laparoscopic robot-assisted pancreas transplant was performed in Italy in 2010
Training and Research: Research and Development; Teaching, training, and Professional Development	Step 8: Plan for a system that supports growth	Italy regularly collaborates in international training programs and research opportunities and participates in international organ exchange schemes

### Enabling Elements

#### Government: Key Legislation

Step 1 Generate legal definitions of living and deceased donation.

Organ donation can be a sensitive subject. A carefully considered legislated definition in common language can reduce miscommunication and protect advocates working in organ donation and transplantation systems. Two important pieces of legislation have shaped the legal acceptance of organ donation and transplantation in Italy. First, Law 458/1967 defined living donation (LD) as a lawful practice. Where a living donor program exists, there is ideally a Living Donor Committee and Donor Advocate to assess guide the decision-making process and evaluate medical safety. Sometime later, Law 578/1993 defined deceased donation (DD) by specifying brain death as an “irreversible loss of all cerebral functions and death certification by neurological (independent council of 3 specialists with a 6 h observation time) or cardiac (20 min with no cardiac activity as shown by electrocardiogram) criteria” (10).

An additional piece of critical legislation was established by Law 91/1999. This law specified the structure of governance for the organ transplant system that included the founding of the CNT and the National Technical Transplant Council, the designation of the Regional Transplant Coordinating Centers, and the definition of the role of hospital procurement coordinators (10). This legislation further legitimized the Italian organ donation system and coordination networks and committed resources toward the newly-developed national program.

#### Building and Maintaining Public Support and Trust in the System

Step 2 Take steps to ensure that altruistic donation and transplantation become part of the national culture and a point of pride.

Positive attitudes reflected by the general public are crucial to the success of any donation and transplant program. Personal stories of organ donations used to treat transplant recipients typically capture the imagination of the public at large. Toward this end, Italy has provided one of the most affecting organ donation stories of all time. In 1994, Nicholas Green was a young American boy on vacation with his family in Southern Italy when he was fatally shot in a failed robbery attempt. His family’s decision to donate his organs and corneas elicited worldwide media attention, including movies and books based on his family’s story. Nicholas’ family received honors from the President of Italy and the Pope (11). As this event occurred during the time in which Italy’s transplant program was just beginning to grow, Nicholas’ story had a profound effect on the Italian public and brought awareness to the somewhat limited number of organs that had been donated in the 1990s (12). Italy experienced a three-fold increase in organ donations following this tragedy (11, 12); “l’Effetto Nicholas” triggered by his family’s donation of his organs after the accident reflects a change in the national consciousness of the Italian population and their understanding of the importance of organ donation.

Since this event, Italy has consistently invested in national and international media campaigns designed to encourage awareness and ongoing discussion of the topic of organ donation and transplantation. The CNT has coordinated a project (COORENOR-2010/2012) and a Joint Action (FOEDUS-2014/2016) that is part of the pan-Europe EUDONORGAN project; a key component of this project is to raise social awareness of organ donation and transplant programs (13). In October 2014, Italy organized the European Organ Donation Day (EODD) in partnership with the Council of Europe. Nationally, the CNT holds annual campaigns, including “Diamo il meglio di Noi” (for organ tissue and cell transplantation) and “Match it now” (for bone marrow transplantation). Two very successful campaigns launched by CNT known as “Salvo e Gaia” and “Ti Voglio Donare” are targeted at primary and secondary school children, respectively. These programs are designed to make certain that discussions of organ donation and transplantation remain part of the normative culture (14). As a result, Italy continues to show favorable and increasingly positive behaviors and attitudes towards organ donation and transplantation (15).

Equity in organ allocation is also of paramount importance to build trust in the system. The CNT provides national indications to assess candidacy for transplant based on several factors (e.g., blood group, antigen compatibility, age, waiting time, among others). These indications are shared by each transplant centre and each region. While general organ allocation is managed regionally, the CNT oversees organ assignment for specialized populations of pediatrics, emergencies, hyperimmune populations, the split liver program, cross-over kidney program, and organ exchanges with foreign countries and organs in surplus from a region (16). The CNT is also responsible to ensure the allocation procedures are met through audits and accreditation (17), which helps maintain a fair transplant system for all.

### Structural Elements

#### The National Transplant Organization (NTO); Infrastructure; Reimbursement of Staff and Facilities

Step 3 Seek out examples of successful programs.

The Italian organ donation and transplantation program is recognized as one of the best adaptations of the Spanish model (18). Elements adopted from the Spanish program include a single NTO that serves as a support agency, a three-level organizational structure with regional centers of excellence (Table 4), and similar transplant coordinator programs (18). The overall healthcare systems of the two countries share common features; this may have facilitated the adoption of a similar approach to organ donation and transplantation. For example, both countries provide universal healthcare and have adequate numbers of acute care beds as well as physicians and nurses. A previous study also compared intensive care unit (ICU) beds per million inhabitants in Spain (66.3) versus Italy (60.4) demonstrated similarities with regional variations with the Tuscany region having one of the highest capacities (73.4) (18). These factors undoubtedly provided favorable conditions that permitted Italy to reproduce many of the components of the Spanish organ donation and transplantation model (18). The variable (as opposed to fixed) schemes used to reimburse transplant activity used in both Italy and Spain serve to incentivize hospitals and (in some instances) healthcare professionals to undertake organ donation and transplant activities, most notably the identification of potential donors (18, 19). This success is reflected in organs donated from 370 hospitals across Italy (10). The Italian experience suggests transformation may be facilitated by starting with a successful plan while recognizing that modifications may be required to adapt it to the needs of a particular country or context.

**TABLE 4:** Structure of the Italian Transplant Network from the ISS website (3).

National transplant network	
National	National Transplant Center (CNT)
	• Authority for the donation and transplantation of organs, tissues and cells
	• Organization of training for transplant specialists
	Permanent technical consultation for transplants
	• Consultative body and prepares the technical and operational guidelines for the donation and transplantation of organs, tissues, and cells
Regional	Regional or Interregional Transplant Centers (CRT)
	• Public structures that coordinate procurement, donation and transplant activities at the regional level and proceed with the assignment of organs
Local	Hospital Coordination
	• Structures and clinical teams that ensure the immediate communication of donor data to the CRT and CNT, coordinate the administrative documents relating the withdrawal operations, take care of relationships with donor families and collect data on transplants
	Withdrawal Facilities
	• Public health facilities where organ, tissue and hematopoietic stem cells are collected for transplantation purposes
	Transplant Structures
	• Public hospitals with authorized transplant teams
	Tissue Institutes
	• Tissue banks to process, conserve, store and distribute human tissues and cells

#### Registries, Databases and Information Technology

Step 4 Create a situation in which it is easy to become a donor.

Soft opt-out consent policies, such as those adopted by Italy, are important but not sufficient to encourage organ donation (20). In a soft opt-out donation consent policy, individuals are presumed to be candidates for organ donation unless specified otherwise by themselves or their families. Italians who have decided on this matter can register with the National Transplant Information System (Sistema Informativo Trapianti [SIT]) (3). This is a combined registry that captures information on positive or negative consent to organ donation. Registration is prompted on multiple occasions and can be made at the office of health authorities, through voluntary donor associations [i.e., Associazione Italiana Donatori Organi (AIDO)], or (since 2013) when first receiving or renewing national identification cards (21, 22). In 2018, 73.2% of registrants listed positive consent, while 26.8% expressed negative consent (26.8%) (21). Southern Italy has a higher refusal rate (40%) in comparison to the best-performing regions in Tuscany and Northern Italy, with a refusal rate of 20% (10).

The CNT is also responsible for keeping a central repository of people awaiting transplant. It also collects, manages, and analyses activities of the transplant centres for statistical, epidemiological and operational purposes using the Transplant Information System. The CNT keeps reports on adverse reactions, ensures traceability of all organs and tissues, and processes all data transmitted from the different regions related to organ donation and transplantation (17).

#### Quality Standards and Continual Quality Improvement

Step 5 Learn from one’s mistakes.

Regulation and quality assurance is of utmost importance. The quality and safety procedures in the Italian transplant system have undergone extensive review after an incident in 2007 in which three patients received organs from a human immunodeficiency virus-positive donor. In response to this error, the CNT organized a formal committee that generated recommendations on safety, quality assurance, and risk management procedures designed to avoid future incidents (23). Each regional transplantation or coordination center now undergoes periodic audits with special attention paid to safety protocols. Extra resources were allocated to support a team of experts from the CNT’s national allocation office that is available at all times for consultation and support. This team includes physicians from the transplantation network, an infectious disease expert, a pathologist, an individual capable of performing resuscitation, and an expert in clinical immunology (23). In practice, the CNT is responsible for issuing the quality regulations and guidelines and conducting safety checks while the regional centers run quality assurance programs (10). The Italian program adheres to international and European standards (for example, 2010/S3/EU, which is the EU directive on the quality and safety of organs for transplantation) and has also taken the lead on several safety-related initiatives *via* international collaborations (i.e., Alliance O) (10, 23).

### Operational Elements

#### Prevention

Step 6 Work to diminish risk factors that may ultimately lead to the need for organ donation.

Many high-income countries are currently experiencing a substantial change in population demographics. Currently, one out of every five Italians are over 65 years of age (5). Prevention of end-organ disease from cardiovascular risk factors is an integral part of any solid organ transplantation system. The prevalence of chronic kidney disease (CKD) is relatively lower in Italy than in other European countries, with an estimated prevalence of 5.7%–12.7% (24–26). By contrast, Italians exhibit comparatively higher rates of CVD and smoking. The most recent CArdiovascular risk in Renal Patients of the Italian Health Examination Survey (CARHES) study estimates the prevalence of CKD in men and women to be 7.5% and 6.5%, respectively, with a higher prevalence of stages 1–2 than 3–5 (21). In 2016, the incidence of renal replacement therapy in Italy was 144 pmp, which was higher than the European average of 132 pmp (27).

Cardiovascular risk factors are also linked to the development of hepatic and end-stage renal disease (ESRD). The prevention of CKD is recognized by the Italian Ministry of Health as a way to mitigate the development of interdependent conditions that contribute to metabolic syndromes (i.e., diabetes, hypertension, and CVD). Primary prevention strategies in Italy are aimed at reducing all cardiovascular-associated metabolic syndromes *via* strategies that include the promotion of exercise and improved dietary choices. Public health campaigns addressing this issue include “Gaining Health: making healthy choices easy” (Prime Ministerial Decree of 4 May 2007) and participation in the World Action on Salt Program (21). Italy has also focused on secondary prevention initiatives that may prevent the transition from early CKD to ESRD, including renal screening and early detection programs in patients who are at risk or are medically frail.

#### Donation and Transplantation

Step 7 Increase donations and transplantations *via* innovative strategies and policies.

Italy is an undisputed leader in DD. However, with the need for organs currently outstripping the supply, no country should ignore opportunities to increase the donor pool or the likelihood of successful transplants.

To increase organ donations and transplantations, Italy’s living donation program requires augmentation. In 2020, only 304 transplants occurred thanks to living donors in comparison to over 3,000 transplants from deceased donation (28). High living donor kidney transplant activity mainly occurred in three centres (Padua and Bari, previously Pisa) whereas living donor liver transplant activity was highest in Palermo, Rome and Milan (28, 29). A consensus conference recommended focusing on the following education activities: increase communication to patients by primary care and community nephrologists, avoid late referrals to specialists, reduce preconceptions about the priority of living donations, increase educational interventions in people with chronic kidney disease and clinicians who deliver dialysis, and remove obstacles to donation (e.g., reducing individual spending and increasing clinical follow up of donors) (29). Certain regions have had difficulties implementing all recommendations, resulting in the recent launch of a pilot project educational campaign in collaboration with the CNT, the Italian Society of Nephrology, and the National Association of Hemodialysis-Transplant Dialysis (Associazione Nazionale Emodializzati-Dialisi Trapianto) in vulnerable geographic areas (30).

Italy has one of the largest split liver transplantation programs worldwide. The CNT instituted a mandatory split liver policy in 2015 in partnership with the Italian College of Liver Transplantation Program (31). Splitting a donated liver expands the available graft pool for pediatric candidates while maintaining sufficient liver tissue for transplantation in adult candidates (32). Ongoing research will determine how to identify candidates that are best suited for this procedure as well as the use of recipient-donor matching procedures to improve outcomes. Italy currently has the most liberal split liver eligibility policy; a recent assessment revealed no increase in morbidity or mortality associated with these protocols (31). Italy is also a leader in research efforts to maximize potential split donations. This effort is aided by a national transplant organization that invests in organ-exchange networks, fosters collaboration between pediatric and adult centers, and standardizes the training of surgeons who perform split procedures (31).

Italy has also become a leader in novel perfusion techniques. This is largely due to national DCD criteria which require a “no-touch” period of 20 min; this is significantly longer than in most other countries that mandate a 5-min interval during which an individual is monitored before the declaration of death. Historically, this has discouraged the use of DCD in Italy due to concerns regarding the possibility that prolonged warmth and ischemia will reduce the quality and viability of the donor graft (14). However, in recent years, Italy has been experimenting with strategies that might circumvent this problem. In 2007, the first pilot project using normothermic regional perfusion (NRP) was performed successfully on a kidney transplant donor. Since that time, Italy has developed protocols to procure healthy organs from uncontrolled DCDs with the potential for prolonged ischemia. Various strategies have been utilized to maintain organ viability and preservation, including machine perfusion, mechanical ventilation, hypothermic oxygenated machine perfusion, and abdominal NRP. Each hospitals funds these novel programs through finances received from their region or may procure additional resources through research or industry partnerships. The use of these novel and innovative perfusion techniques is balanced by the culture of safety in Italy. For example, the Italian Society of Organ and Tissue Transplantation (Società Italiana Trapianti d’Organo [SITO]) has issued cautious position papers regarding the use of machine perfusion in liver transplantation. Although these preservation techniques are still under development, the initial results are promising and suggest that this may ultimately be a useful way to expand the available organ donor pool in Italy (33).

More than 40 Italian hospitals have transplant programs, including 41 kidney, 22 liver, 11 lung, and 16 heart-dedicated programs. There are also pilot programs dedicated to bowel, pancreatic islet, hand, face, and uterus transplants (10). The use of robotic assistance for transplant surgery was also pioneered in Italy (34). The first laparoscopic robot-assisted pancreas transplant was performed in 2010 at Pisa University Hospital (35). The Milan criteria for liver transplantation for hepatocellular carcinoma is another example of the ongoing research and innovation activities currently taking place in Italy. Italy has also been a leader in the clinical practice and development of protocols for the use of organs with donor or recipient infections, including human immunodeficiency virus (HIV), Hepatitis C and most recently, COVID-19 (36, 37). The innovations introduced in Italy benefit the transplant program by improving both quality and efficiency, as well as highlighting the potential for better outcomes. These efforts have helped to establish transplantation as a pioneering field in Italy and thus to focus attention and acquire resources from national health authorities.

### Training and Research

#### Teaching, Training and Professional Development

Step 8 Plan for a system that supports growth.

The CNT values ongoing development. Many opportunities for sustaining progress are undertaken in conjunction with international partners. Transplant coordinators receive bi-annual training courses organized by the CNT (13). Additional coursework that focuses on organ coordination and improving communication skills are held regionally (13). The CNT also hosts international donor-training courses in conjunction with the University of Padua and Veneto Regional Transplant Center that are designed to help transplant surgeons gain mastery in organ procurement. This coursework is endorsed by the European Society for Organ Transplantation (ESOT) and the European Union of Medical Specialists (UEMS) and is provided free of charge to successful applicants (13).

The pioneers of the Italian Transplant Network established close relationships with their Spanish colleagues early on during the development of the transplant system. This collaboration eventually developed into the South Alliance for Transplant (SAT) network which facilitates knowledge sharing, training collaborations, and expansion of the donor pool. In 2018, Spain and Italy collaborated to perform the first live donor kidney exchange in Southern Europe (38).

In addition to promoting national research advances in the field of organ donation and transplantation, the CNT is also actively involved in European and international research projects and registries. The CNT engaged in bi- and multilateral agreements for international organ exchanges beginning in the early 2000s (10). In collaboration with the World Health Organization, the CNT launched Project NOTIFY, which is “a global interface for the vigilance and surveillance of substances of human origin (39).” This initiative is designed to document adverse events and reactions to improve donor and recipient safety. Italy is one of the few countries that publishes all transplant data as part of the effort to maintain full transparency in organ allocation and management (40). This process is facilitated by a fully-functional registry that collects activity data on organ donation, retrieval, and transplantation. Data focused on follow-up of all transplant recipients are also collected. The process also includes a reporting system that is used to document serious adverse events and reactions (3).

## Discussion

This paper aimed to highlight our current understanding of the critical components contributing to the success of the Italian organ donation and transplantation program. This objective was achieved by referring to a system-level conceptual framework and validating our findings by seeking input from subject matter experts. The results are presented as eight steps (summarized in Table 3) that were designed to demonstrate the most important aspects of a successful organ donation and transplantation program. However, this presentation may to some degree conceal the true complexity involved in building a successful program.

In addition to these eight steps, two additional important points arose from this case study. First, the messages conveyed when constructing and implementing a transplant system must be simple and comprehensible. A common language and direction are vital when communicating with individuals from diverse disciplines and backgrounds, including individuals tasked with governing a transplant system and extending to those who carry out donor activities at the local level. Second, each step must be supported by a broad range of sustainable actions, staffing, initiatives, and resources. For example, while altruistic donation and transplantation became part of the national culture in Italy because of an initial emphasis on a tragic story, constant repetition through campaigns and decades of positive relationships with the media will be needed to maintain transparency and goodwill throughout. It is also important to recognize that many of these steps are interconnected. For example, organ donations cannot become accepted as part of the normative culture in the absence of critical infrastructure, including databases and registries that simplify the processing of becoming a donor.

Although alluded to throughout this paper, within the successes of the Italian organ donation and transplantation system, there are notable regional differences whereby the system must be improved for equity and efficiency gains. These activity differences by region are substantial as reported by the 2020 Annual Activity Report (28). For example, the rate of effective donors was highest in the Valle d’Aosta region (47.7 pmp) and lowest in the Basilicata region (5.3 pmp). Differences in donation refusal rates and ICU capacity are briefly mentioned, but there are also variations in procurement rates, number of transplant programs for all types of tissues, consent for on donation, among others (28). Of concern, northern regions appear to consistently have better indicators for all organ donation and transplant activities than southern regions. These regional differences are not limited to transplant activities; geographical inequity has been a longstanding issue in Italy with the more affluent north leading the way in bed capacity, advanced medical equipment, health and community care resources and citizen health outcomes (5, 6). While a thorough analysis of interregional inequity is outside of the scope of this paper, for organ donation and transplantation, these inequities result in some regions being unable to effectively apply the best practice standards recommended by the CNT and this impacts accessibility of transplants for specific populations.

As there are many limitations to the targeted literature review methodology, the scope of this paper must be clear. At each step of this process, from the methods through the data analysis, our purpose was to identify lessons from the Italian transplant system that could be applied to new and developing national organ donation and transplantation systems. A targeted literature review reinforced by expert opinions was deemed to be the best approach to achieving this aim. However, we recognize that this approach to data collection and analysis is prone to bias and we appreciate the uncertainty regarding our conclusions and the ability to apply them in other unique settings. Despite these issues, we believe that this approach was the most feasible way to assemble information that spanned multiple different sectors, including (but not limited to) clinical medicine, public policy, ethics, and media relations. Taken together, our findings provide a starting point for future research into the development of organ donation and transplantation programs.

Notably, the transplant literature rarely discusses the outcomes of transplant programs (i.e., statistics associated with donations and transplants) as the consequence of well-conceptualized and carefully developed and implemented coordination efforts at the systemic level. This paper, together with the other case reports included in this series (41–45) will to some extent address this knowledge gap as they provide new insights on how one might conceptualize the elements required by a developing national organ donation and transplantation system.

## Data Availability

The original contributions presented in the study are included in the article/supplementary material, further inquiries can be directed to the corresponding author.
